# Early pregnancy-induced transcripts in peripheral blood immune cells in *Bos indicus* heifers

**DOI:** 10.1038/s41598-020-70616-8

**Published:** 2020-08-13

**Authors:** Cecilia Constantino Rocha, Sónia Cristina da Silva Andrade, Gabriela Dalmaso de Melo, Igor Garcia Motta, Luiz Lehmann Coutinho, Angela Maria Gonella-Diaza, Mario Binelli, Guilherme Pugliesi

**Affiliations:** 1 Department of Animal Reproduction, School of Veterinary Medicine and Animal Science, University of São Paulo, Pirassununga, São Paulo Brazil; 2 Department of Genetics and Evolutionary Biology, University of São Paulo, São Paulo, São Paulo Brazil; 3 Laboratory of Animal Biotechnology, School of Agriculture Luiz de Queiroz, University of São Paulo, Piracicaba, São Paulo Brazil; 4 North Florida Research and Education Center, Institute of Food and Agricultural Sciences, University of Florida, Marianna, FL USA; 5 Department of Animal Sciences, University of Florida, Gainesville, FL USA

**Keywords:** Animal physiology, Transcriptomics

## Abstract

Immune cells play a central role in early pregnancy establishment in cattle. We aimed to: (1) discover novel early-pregnancy-induced genes in peripheral blood mononuclear cells (PBMC); and (2) characterize the temporal pattern of early-pregnancy-induced transcription of select genes in PBMC and peripheral blood polymorphonuclear cells (PMN). Beef heifers were artificially inseminated on D0 and pregnancies were diagnosed on D28. On D10, 14, 16, 18, and 20, blood was collected for isolation of PBMC and PMN from heifers that were retrospectively classified as pregnant (P) or non-pregnant (NP). PBMC samples from D18 were submitted to RNAseq and 220 genes were differentially expressed between pregnant (P) and non-pregnant (NP) heifers. The temporal abundance of 20 transcripts was compared between P and NP, both in PBMC and PMN. In PBMC, pregnancy stimulated transcription of *IFI6*, *RSAD2*, *IFI44*, *IFITM2*, *CLEC3B*, *OAS2, TNFSF13B, DMKN* and *LGALS3BP* as early as D18. Expression of *IFI44*, *RSAD2*, *OAS2, LGALS3BP, IFI6* and *C1R* in PMN was stimulated in the P group from D18. The novel early-pregnancy induced genes discovered in beef heifers will allow both the understanding of the role of immune cells during the pre-attachment period and the development of technologies to detect early pregnancies in beef cattle.

## Introduction

In cattle, pregnancy success depends on the maintenance of a functional corpus luteum (CL) beyond the time of luteolysis, which normally occurs between days 15 and 18 of the estrous cycle^1,2^. The CL maintenance in ruminants occurs in response to interferon-τ (IFN-τ) secreted by the conceptus^3^. The INF-τ glycoprotein binds to its receptors and consequently inhibits the pulsatile secretion of prostaglandin F2α (PGF2α) from the endometrium, preventing luteolysis^4^. Consequently, the CL remains active and secretes progesterone (P4) in concentrations sufficient for the establishment of pregnancy^1^. Success in this sequence of events determines the outcome of pregnancy; however, as many as 50% of pregnancies fail until day 17 after artificial insemination (AI)^5^.

The mechanisms that mediate embryo survival and death are incompletely understood, but the maternal immune system plays an important role in embryo development during the pre-attachment period^6^. A functional connection between the maternal immune system and the developing embryo is IFN-τ. The day-4 bovine embryo is already capable of synthesizing IFN-τ, which regulates the local immune environment in the oviduct^7^. In addition, the in vitro development of bovine embryos from morula (day-5) to blastocyst stage (day-9), starts signaling the uterine epithelial and immune cells in co-culture to modulate the anti-inflammatory response mediated by IFN-τ^8^. These changes contribute to conceptus growth and maternal immune modulation to prevent conceptus rejection^9^. Therefore, an immunological crosstalk between embryo and immune cells exist locally in the uterus, however, how those signals influence the immune cells is currently unclear.

Studies in ruminants during the last decades have reported that the IFN-τ up regulates the expression of interferon stimulated genes (ISGs) during early pregnancy in various tissues, such as the endometrium^10^, luteal cells^11^, liver^12^ peripheral blood mononuclear cells (PBMC) and peripheral blood polymorphonuclear cells (PMN)^13^. The transcriptional profile of classic ISGs, such as Ubiquitin like modifier 15 (*ISG15*), 2′-5′-Oligoadenylate synthetase 1 (*OAS1)*, MX dynamin like GTPase 1 (*MX1)* and MX dynamin like GTPase 2 (*MX2*) is closely associated with the IFN-τ secretion by the conceptus. Indeed, transcription usually increases from day 15 post-AI, reaches a peak on day 20 and reduces from day 22 on^14–16^. These findings suggested that IFN-τ may modulate the immune system during early pregnancy. Such information was used in recent studies that established that ISGs are diagnostic markers of pregnancy between days 18 and 20 days post-AI^14,15^.

In this context, early detection of pregnancy loss after AI is critical for dairy and beef cattle operations. The method to diagnose pregnancy that is used mostly in the beef industry is based on the ultrasonographic visualization of a viable embryo between 28 and 35 days after breeding^17^. Although the use of clean-up bulls after a timed-AI (TAI) is still the most used strategy in beef operations, the use of resynchronization programs based on efficient detection of pregnancy and exposure of non-pregnant females to a second TAI has increased in the last decade in beef and dairy operations^18^. Thus, in TAI programs the interval between two TAI is usually 32–40 days. For this reason, development of earlier diagnostic methods (≤ 20 days post-TAI) are desirable, because they would make possible to shorten the interval between subsequent TAIs, leading to improved reproductive efficiency^19,20^. Recently, earlier resynchronization strategies have been developed based on the detection of structural luteolysis using color Doppler ultrasonography to evaluate the luteal blood perfusion^14,21,22^. Although this method can be used as early as day 20 post-TAI and the occurrence of false-negative results are minimal, the presence of the conceptus is not detected and there is a 15–25% of false-positive diagnostics in dairy cows and beef heifers^23,24^.

A greater understanding of the role of immune cells during early pregnancy may result in improvements in the practical use of ISG expression as a tool to diagnose early pregnancies in cattle. For example, one study that compared ISG expression between PBMC and PMN reported that PMN may have an earlier response to IFN-τ secretion^13^, but that requires confirmation. Furthermore, the accuracy of using the expression of the classic ISG transcripts on PBMC for pregnancy detection on day 20 post-TAI is only about 60–83% in beef cows^14,25^. Hence, one potential strategy to improve this accuracy and to better understand the immune physiology during early pregnancy is the discovery of novel transcripts stimulated by the presence of a viable conceptus and use them as pregnancy markers in PBMC or PMN.

Therefore, we aimed with the present study: (1) to discover novel potential pregnancy markers in cattle through transcriptomic analysis using PBMC on day 18 post-AI in beef heifers; and (2) to evaluate the pregnancy-induced mRNA profile of those novel markers in PBMC and PMN during the first weeks post-AI. We hypothesized that newly-identified transcripts, other than the classic ISGs reported in immune cells, are differentially expressed between pregnant and non-pregnant beef heifers. We anticipate that such differences will serve as the basis for the selection of novel pregnancy markers in bovine immune cells before day 20 post-TAI. Furthermore, clues on novel mechanisms through which the conceptus signals its presence to the maternal system and modulates immune cell function will be gleaned from the current results.

## Results

The probabilities (significance or approached significance) for a group effect, a time effect and the group-by-time interaction were shown in the figures, and the probabilities for differences in discrete endpoints are given in the text.

### Study 1

**Figure 1:**
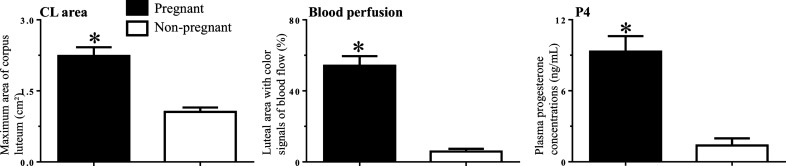
Mean ± SEM for CL area, CL blood perfusion and plasma P4 concentrations on D18 post-TAI of the pregnant and non-pregnant heifers select for RNAseq (6/group). *Means indicate differences (*P* < 0.05) between groups.

The pregnancy rate on D28 post-TAI in the heifers that ovulated (n = 21) to the protocol was 43% (9/21). For the heifers selected for the transcriptomic analyses (n = 6/group), as expected, a greater CL area (*P* = 0.0002) blood perfusion (*P* < 0.0001) and plasma P4 concentrations (*P* = 0.003) on D18 was observed in the P group than in the NP group (Fig. 1).

**Figure 2:**
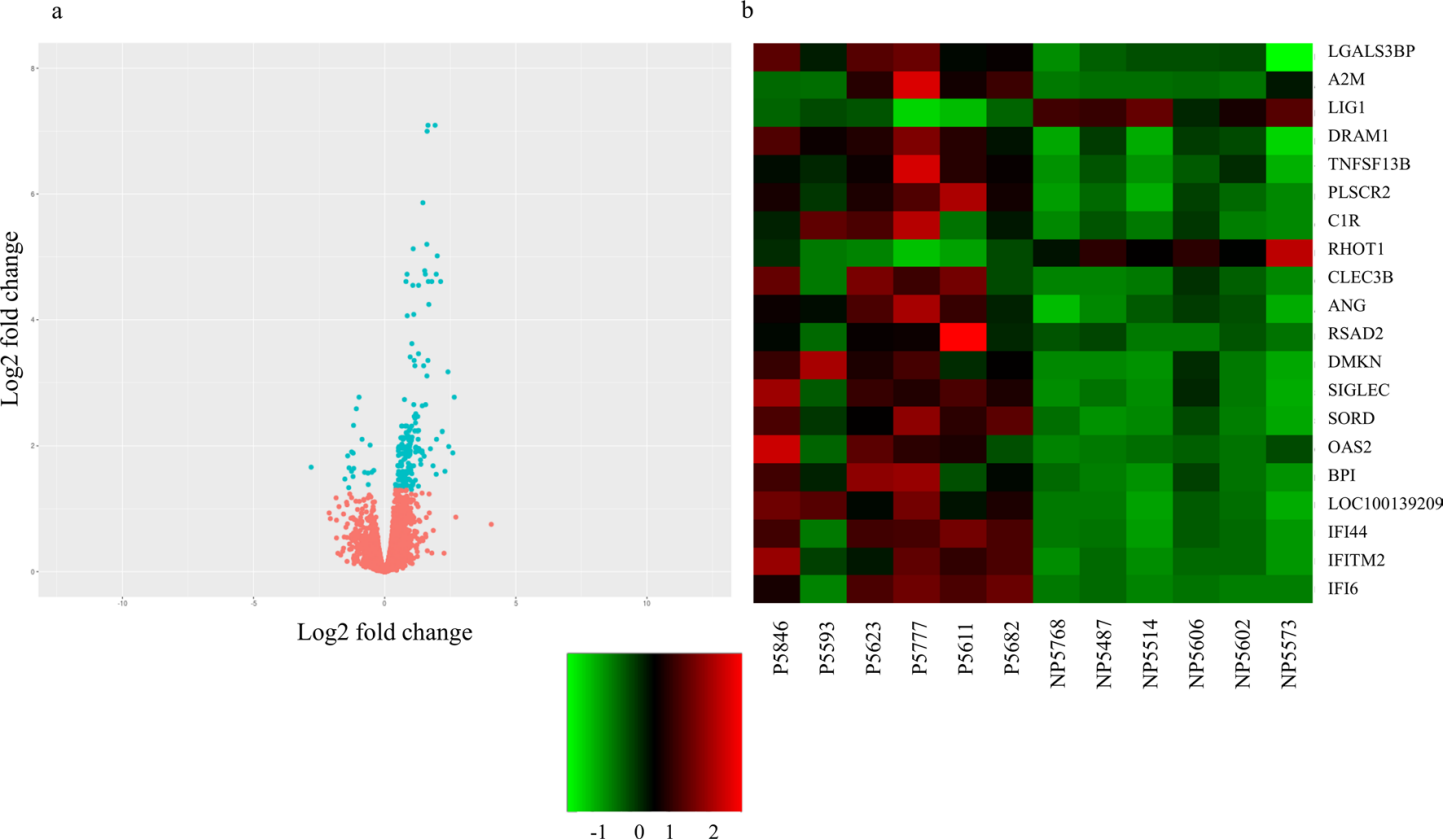
**a** Volcano plot showing pregnant (P; n = 6) and non-pregnant (NP; n = 6) gene expression, in terms of the differentially expressed genes (FDR < 0.05). While **b** heat maps constructed showing the 20 genes selected using the criteria of overlap absence and highest log fold change, for the comparison between pregnant (P; n = 6) and non-pregnant (NP; n = 6) heifers. The colors in the map display the relative standing of the reads count data; Green indicates a count value that is lower than the mean value of the row while red indicates higher than the mean. The shades of the color indicate distance from each data point to the mean value of the row. Columns represent individual samples of P and NP group.

The RNAseq produced a total of ~ 240 million reads with an average of 20 million reads for each sample. Six biological replicates were analyzed for each group with the reads ranging from 17 to 21 million per sample after filtering. After use STAR, approximately ~ 90% of the total reads uniquely mapped to the reference genome, excluding also reads that aligned ambiguously. After applying the variance and minimal value of base Mean filtering, a total of 13,434 genes were included in the differential expression analysis. A total of 220 out of the 13,434 analyzed genes showed differential expression, of which, 200 were up-regulated on P group and 20 down-regulated on P group (Fig. 2a) (FDR < 0.05). All read sequences (raw files and processed files) and an overview of this data has been deposited in NCBI’s Gene Expression Omnibus (GEO) and is accessible through GEO Series accession number GSE136102.

After excluding seven out of 220 DEGs that were classic ISGs in immune cells (*ISG15*, *MX1*, *MX2*, *OAS1Y*, *OAS1X*, *OAS1Z and MIC1*), we selected 20 DEGs for further evaluation, based on the two criteria: (1) DEGs that did not present overlap in the RNAseq TMM values between NP and P groups and (2) the DEGs with the highest log fold change (logFC) values (Fig. 2b). Eleven DEGs were selected based on the first criterion: Angiogenin (*ANG*), Dermokine (*DMKN*), DNA Damage Regulated Autophagy Modulator 1 (*DRAM1*), Interferon Induced Transmembrane Protein 2 (*IFITM2*), DNA Ligase 1 (*LIG1*), Galectin 3 Binding Protein (*LGALS3BP*), LOC100139209, Phospholipid Scramblase 2 (*PLSCR2*), Ras Homolog family member T1 (*RHOT1*), Sorbitol Dehidrogenase (*SORD*) and TNF superfamily 13b (*TNFSF13B*). Using the second criterion, the other nine DEGs analyzed were: Alpha-2-Macroglobulin (*A2M*), Bactericidal Permeability Increasing Protein (*BPI*), Complement C1r (*C1R*), C-type Lectin Domain family 3 member B (*CLEC3B*), Interferon Alpha protein 6 (*IFI6*), Interferon Alpha protein 44 (*IFI44*), 2′-5′-Oligoadenylate Sinthetase 2 (*OAS2*), Radical S-Adenosyl Methionine Domain Containing 2 (*RSAD2*) and Sialic Acid Binding Ig-like lectin 1 (*SIGLEC1*) (Table 1).

**Table 1:** List of the genes selected using the criteria of: highest log fold change (FC) (first column) and absence of overlap criteria (third column) with their respectively FC value.

Without overlap	Log FC value	Greatest Log FC	Log FC value
*IFITM2*	1.9224	*C1R*	2.4414
*LOC112448753*^a^	1.6828	*SLCO2B1*	2.4124
*SORD*	1.6632	*A2M*	2.2973
*DMKN*	1.6092	*CLEC3B*	2.1951
*LOC100139209*	1.6062	*OAS2*	2.1323
*LOC100852090*^a^	1.4337	*IFI6*	1.6506
*LOC782062*^a^	1.4317	*SIGLEC1*	1.6480
*FRMD4A*^a^	1.2734	*RSAD2*	1.5611
*TNFSF13B*	1.1912	*BPI*	1.5484
*ANG*	1.1761	*IFI44*	1.4556
*LOC112443203*^a^	0.9729		
*DRAM1*	0.8653		
*PLSCR2*	0.5459		
*CASP1*^a^	0.5245		
*SLC1A5*^a^	0.5199		
*LGALS3BP*	0.4285		
*LIG1*	− 0.4165		
*RHOT1*	− 0.5568		

The negative FC value means genes down-regulated on pregnant group, while positive FC value means genes up-regulated on pregnant group.

^a^Means genes that were found with the criteria but not validated by qPCR.

Although the main purposes of the herein study is to identify novel transcripts in immune cells for using as a maker for pregnancy detection, to understand the functional implication of DEGs between P and NP groups, we performed a GO enrichment analysis. The GO analysis with the up-regulated DEGs on P group resulted in 42 chart records overrepresented for biological process category, 17 for cellular component category and 19 for molecular function category (Supplemental File 1). The same DEGs were submitted to KEGG pathway enrichment analysis and 19 overrepresented KEGG domains were identified. There were no overrepresented significant charts for the DEGs downregulated on P group in the GO enrichment and KEGG pathway analysis (Supplemental File 2).

### Study 2

**Figure 3:**
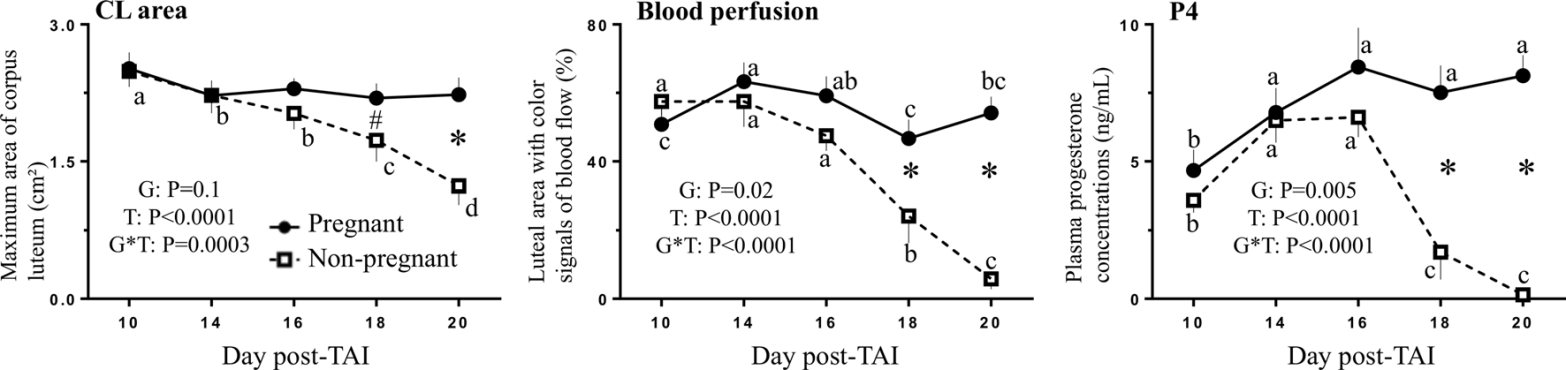
Mean ± SEM for CL area, CL blood perfusion and plasma P4 concentrations from days 10 to 20 post-TAI of pregnant and non-pregnant heifers (n = 6/group). Probabilities are shown for significant main effects of group (G) and time (T) and the interaction for group-by-time (G*T). *^#^Means indicate differences (*P* ≤ 0.05 and *P* ≤ 0.1 respectively) between groups in specifics days. ^abcd^Means without a common letter within a group indicate differences (*P* < 0.05) between days.

The main effects of time and group and the interaction of group-by-time were significant for CL area, CL blood perfusion and plasma P4 concentrations (Fig. 3). The interaction of group-by-time represented a greater (*P* < 0.05) or approached greater (*P* ≤ 0.1) CL area and blood perfusion and plasma P4 concentrations on D18 and D20 in the P group than in the NP group.

**Figure 4:**
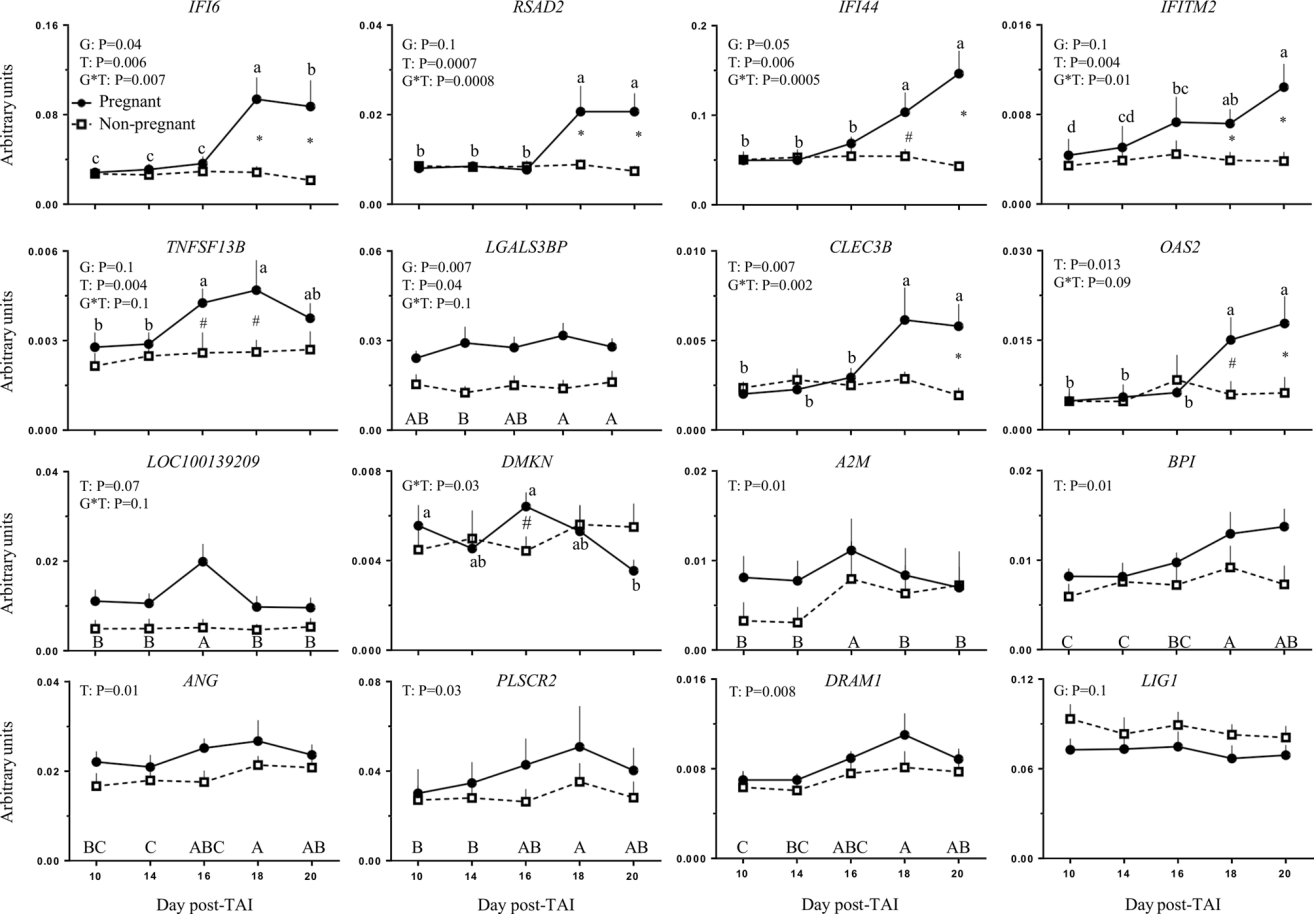
Mean ± SEM for relative expression by qPCR on PBMC of the genes with significant effects during statistical analysis: *IFI6*, *RSAD2*, *IFI44*, *IFITM2*, *TNFSF13B*, *LGALS3BP*, *CLEC3B*, *OAS2*, *LOC100139209*, *DMKN*, *A2M*, *BPI*, *ANG*, *PLSCR2*, *DRAM1* and *LIG1*, from days 10 to 20 post-TAI of pregnant and non-pregnant heifers (6/group). Probabilities are shown for significant main effects of group (G) and time (T) and the interaction for group-by-time (G*T). *^#^Means indicate differences (*P* ≤ 0.05 and *P* ≤ 0.1 respectively) between groups in specifics days. ^abcd^Means without a common letter within a group indicate differences (*P* < 0.05) between days. ABCD Means difference between days in all groups (*P* < 0.05).

Transcription of the 20 genes selected was analyzed in PBMC by qPCR from D10 to D20 after TAI. For *IFI6*, *RSAD2*, *IFI44*, *IFITM2*, *TNFSF13B* and *LGALS3BP,* a significance (*P* < 0.05) or approached significance (*P* ≤ 0.1) effects of time and group and the interaction of group-by-time were observed. The interaction for *IFI6*, *RSAD2*, *IFI44* and *IFITM2* (Fig. 4) reflected that initially the transcript levels start similar between P and NP group, becoming significantly (*P* < 0.05) different in the P group between D16 and D18, resulting in about twofold change greater expression on D18 and D20 in the P group than the NP group. For *TNFSF13B* (Fig. 4), however, the significant (*P* < 0.05) increase on P group was between D14 and D16, resulting in approaching (*P* < 0.1) greater expression (1.5-fold change) on D16 and D18 in the P group than in the NP group. Exception is *LGALS3BP,* where no differences between P and NP groups within a day were observed. Therefore, a significance group effect indicated that the relative expression of *LGALS3BP* was always 50% greater from D10 to D20 in the P group than in the NP group, while a significant time effect reflected a greater expression on D18 and D20, regardless the group (Fig. 4).

For *CLEC3B*, *OAS2* and LOC100139209 (Fig. 4), the main effect of time and the interaction of group-by-time were significant (*P* < 0.05) or approached significant (*P* ≤ 0.1). The interaction for *CLEC3B* and *OAS2* reflected that initially the transcript levels start similar between P and NP, becoming significant (*P* < 0.05) different in the P group between D16 and D18, and resulted in about 1.5–2.5-fold change greater in the P group than the NP group on D18 for *OAS2* and on D20 for both genes. While no effects were found when the interaction for LOC1001139209 was explored, the time effect reflected a significant increased (*P* < 0.05) expression on D16 followed by a decrease on D18, regardless the group (*P* < 0.05). For *DMKN* (Fig. 4), only an interaction of group-by-time was detected, which reflected 0.5-fold change of decrease (*P* < 0.05) between D10 and D14 followed by a similar increase (1.5) (*P* < 0.05) on D16 and a second decrease (*P* < 0.05) on D18 in the P group.

Although a significant main effect of group or group-by-time interaction was not detected for *A2M*, *BPI*, *ANG*, *PLSCR2* and *DRAM1* (Fig. 4), a significance (*P* < 0.05) time effect was detected. For *A2M*, the highest (*P* < 0.05) relative expression value was on D16, regardless of the group (P or NP); while for *BPI*, *ANG*, *PLSCR2*, and *DRAM1*, was on D18. For *LIG1* (Fig. 4), only an approached significance (*P* ≤ 0.1) effect of group was observed, as indicated by about 1.5-fold change greater expression in the NP group than in the P group. For *SIGLEC1, C1R, SORD* and *RHOT1* (Supplemental File 3), no significance effects of group, time or interaction of group-by-time were detected.

**Figure 5:**
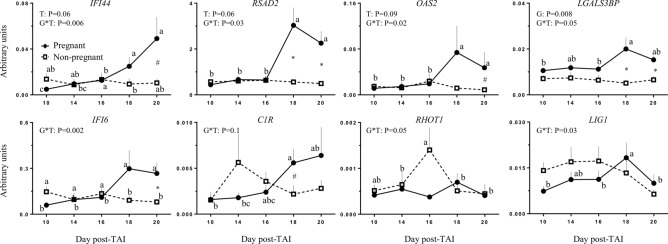
Mean ± SEM for relative expression by qPCR of all genes with significate effects during statistical analysis on PMN: *IFI44*, *RSAD2*, *OAS2*, *LGALS3BP*, *IFI6*, *C1R*, *RHOT1*, *LIG1* from days 10 to 20 post-TAI of pregnant and non-pregnant heifers (n = 6/group). Probabilities are shown for significant main effects of group (G) and time (T) and the interaction for group-by-time (G*T). *^#^Means indicate differences (*P* ≤ 0.05 and *P* ≤ 0.1 respectively) between groups in specifics days. ^abcd^Means without a common letter within a group indicate differences (*P* < 0.05) between days.

For the nine genes evaluated on PMN, a significance (*P* < 0.05) or approached significance (*P* < 0.1) effects of time or group and interaction of group-by-time were observed for *IFI44*, *RSAD2*, *OAS2* and *LGALS3BP* (Fig. 5). The interaction reflected that initially the transcript levels start similar between the P and NP groups, becoming significantly different in the P group between D16 and D18, and resulted in about 2–2.5-fold change greater expression on D18 for *RSAD2* and *LGALS3BP* and on D20 for these four genes in the P group than in the NP group. For *IFI6*, *C1R*, *RHOT1* and *LIG1* (Fig. 5), only a significance (*P* ≤ 0.05) or approached significance (*P* ≤ 0.1) interaction of group-by-time was detected. For *IFI6* and *C1R* the interaction mainly reflected an increased expression in the P group with twofold change greater expression on D20 for *IFI6* and on D18 for *C1R* in the P group than in the NP group. For *RHOT1* and *LIG1* only a 1.5–2.5-fold change greater expression was observed in the P group on D16 for *RHOT1* and on D18 for *LIG1* compared to D10. For *IFITM2* (Supplemental File 4), no significance effects of group, time or interaction of group-by-time were detected.

When the expression of the genes affected by pregnancy status or with an interaction of group-by-time was analyzed according to the relative abundance between D18 and D10 post-TAI (Table 2), a greater (*P* < 0.05) or approaching greater (*P* ≤ 0.1) relative transcript abundance (difference between D18 and D10 and D18/D10) in *IFI6*, *IFI44*, *RASD2*, *OAS2*, *LGALS3BP*, *IFITM2* and *CLEC3B* was detected in P versus NP heifers for PBMC samples. For PMN, a greater (*P* < 0.05) or approaching greater (*P* ≤ 0.1) relative transcript abundance between D18 and D10 was detected in *IFI6*, *IFI44*, *RASD2* and *LGALS3BP* heifers, but not for *OAS2* in P versus NP. In addition, when the relative expression between D18 and D10 in the P heifers was compared between PBMC and PMN, a greater (*P* < 0.05) ratio in *RASD2* and an approaching greater (*P* ≤ 0.1) ratio in *IFI44* were observed on PMN than PBMC.

**Table 2:** Mean ± SEM difference and ratio of the relative expression of genes between days 10 and 18 between pregnant (P) and non-pregnant (NP) on *Study 2*.

Endpoint	PBMC			PMN		
	Pregnant	Non-pregnant	*P* value	Pregnant	Non-pregnant	*P* value
**IFI6**						
Difference between D18 and D10^1^	0.06 ± 0.02	0.001 ± 0.006	0.006	0.2 ± 0.1	− 0.05 ± 0.02	0.03
D18/D10^2^	3.4 ± 0.6	1.1 ± 0.2	0.007	5.6 ± 1.3	0.6 ± 0.2	0.003
**RSAD2**						
Difference between D18 and D10	0.01 ± 0.005	0.0003 ± 0.001	0.02	2.6 ± 0.8	− 0.007 ± 0.1	0.007
D18/D10	2.4 ± 0.4^B^	1.1 ± 0.1	0.01	10.4 ± 3.1^A^	1.1 ± 0.2	0.01
**IFI44**						
Difference between D18 and D10	0.06 ± 0.02	0.004 ± 0.006	0.01	0.02 ± 0.009	− 0.004 ± 0.001	0.02
D18/D10	2.5 ± 0.4^Y^	1.1 ± 0.2	0.01	9.6 ± 3.1^X^	0.8 ± 0.4	0.02
**OAS2**						
Difference between D18 and D10	0.01 ± 0.004	0.001 ± 0.002	0.08	0.06 ± 0.04	− 0.003 ± 0.002	0.1
D18/D10	4.4 ± 1.1	1.8 ± 0.5	0.05	4.9 ± 1.8	1.4 ± 0.8	0.1
**LGALS3BP**						
Difference between D18 and D10	0.008 ± 0.002	− 0.001 ± 0.003	0.04	0.009 ± 0.006	− 0.002 ± 0.001	0.07
D18/D10	1.3 ± 0.09	0.9 ± 0.1	0.04	2.1 ± 0.5	0.7 ± 0.2	0.03
**IFITM2**						
Difference between D18 and D10	0.004 ± 0.001	0.0005 ± 0.0006	0.03	–	–	
D18/D10	2.6 ± 0.5	1.3 ± 0.3	0.07	–	–	
**TNFSF13B**						
Difference between D18 and D10	0.002 ± 0.0005	0.0005 ± 0.0003	0.04	–	–	
D18/D10	1.6 ± 0.1	1.4 ± 0.2	0.4	–	–	
**CLEC3B**						
Difference between D18 and D10	0.004 ± 0.001	0.0005 ± 0.0004	0.04	–	–	
D18/D10	3.1 ± 0.7	1.3 ± 0.3	0.03	–	–	
**DMKN**						
Difference between D18 and D10	− 0.0002 ± 0.001	0.0011 ± 0.001	0.4	–	–	
D18/D10	1.09 ± 0.2	1.4 ± 0.2	0.3	–	–	

The *P* value means significant differences between P and NP in the same cellular type. Superscript letters in the same line indicate significant (^AB^) or approached (^XY^) differences between the immune cell types of pregnant heifers.

^1^The difference was calculated for each individual relative expression on day 18 and subtracted of the same value on day 10.

^2^The individual relative expression of each animal on day 18 was contrasted with the same animal on day 10.

## Discussion

In cattle, the early pregnancy period is associated with paracrine conceptus signaling by IFN-τ to the endometrium, which modulates the immune responses. In addition, endocrine actions of IFN-τ have been proposed to affect several cell and tissues such the immune cells. In the herein study we described novel DEGs in the PBMCs and PMNs between P and NP heifers during the critical period of conceptus signaling and survival. Determination of the DEGs expression profile on immune cells during the first weeks of pregnancy provides the opportunity to further understand the immune modulation of the conceptus-originated stimuli on peripheral immune cells during the preimplantation period. Also, this effort prospected the identification of novel pregnancy markers that can be used to develop an earlier pregnancy detection method in cows based on indirect recognition of conceptus signaling. The development of a novel pregnancy diagnosis method before day 20 of pregnancy has a great impact compared to the traditional method on day 28–30 and a potential improvement on novel approaches to shorten the time from AI to resynchronization and rebreeding.

Our hypothesis that novel transcripts beyond the classic ISGs reported in immune cells would be differently expressed between P and NP beef heifers was supported. Only seven out of the 200 DEGs up-regulated in P heifers were classic ISGs already reported in immune cells. Thus, we were able to select DEGs that are ISGs reported for the first in immune cells (*IFI44*, *IFI6* and *OAS2*), genes not previous related to pregnancy establishment (*RHOT1*, *LIG1*, *DMKN* and *DRAM1*) and even non-named genes like LOC100139209. In addition, the classic ISGs, *ISG15* and *OAS1X* genes were in the top 10 transcripts, up-regulated in P heifers of our results, but with overlapping in TMM values between P and NP samples. This reinforces that the use of these known ISGs as markers for pregnancy diagnosis may result in a high rate of false negative, as recently reported in PBMC and PMN on day 20 of pregnancy in beef cattle^14,25^.

Despite the high number of DEGs found on RNAseq, when we used the criterion absence of overlap on TMM results to find novel markers, fewer candidates were detected. The presence of a greater number of DEGs without overlapping could have occurred if we had compared samples from pregnant animals and non-inseminated animals; however, our main goal was to find pregnancy markers for use as a method to detect pregnancy in inseminated animals from commercial farms. In addition, the use of a group without breed or insemination could has biased the finding of new markers for pregnancy success, as the occurrence of early pregnancy loss before days 18 after TAI can reach 40–50% in cattle^5^. In this regard, we found a difference in CL function and progesterone concentrations between P and NP heifers on D18. Despite the P4 is crucial to the pregnancy maintenance^26^, the expression of DEGs detected on D18 could have been influenced by the conceptus presence or P4 concentrations. Therefore, we believe that the present experimental design allowed the discovery of novel genes stimulated only by a viable conceptus.

The hypothesis that the profile of the DEGs would allow the selection of novel pregnancy markers in bovine immune cells before day 20 post-TAI was also supported. Five genes (*IFI6*, *IFI44*, *RASD2*, *OAS2* and *LGALS3BP*) evaluated in both types of immune cells, have a similar increased expression profile. Other three genes were up regulated before D20, but the different profiles between the immune cell types may represent greater responsiveness to the conceptus presence for *C1R* and *RHOT1* on PMN and for *IFTM2* on PBMC. In this regard, during early pregnancy the main immune cell type attracted by endometrium is the monocyte^27^. But, previous studies^13,28^ suggested that PMN may have an earlier response to INF-τ secretion. Also, Toji et al.^29^ indicated that PMNs are more sensitive to IFN-τ and that the shorten lifespan (few hours) of neutrophils, may impact on the PMN sensitivity to IFN-τ compared to other immune cell types. In this regard, the IFN-τ acts on endometrial cells by Janus kinase-signal transducer and activator of transcription (JAK/STAT) pathway^30^, but the intracellular mechanisms of IFN-τ on immune cells is not well described.

The profile of several genes on both immune cell types followed the expected IFN-τ secretion during trophectoderm expansion. That is, the profile expression of *IFI6*, *IFI44*, *RSAD2*, *OAS2*, *IFITM2* and *CLECL3B* on PBMC and of *IFI6*, *IFI44*, *RSAD2* and *OAS2* on PMN, which increases from day 15 to 16 post-AI and reaches a peak on about day 20^16,31^. In addition, the expression profile observed for these genes corroborates with expression of the classic and most studied ISGs in immune cells (*ISG15*, *MX1*, *MX2*)^14,15^. Therefore, the reported antiviral response mediated by type I interferon (IFN) on the expression of *IFI6*, *IFI44*, *RSAD2*, *OAS2* and *IFITM2*^32–34^ indicates that these genes are also stimulated by the conceptus through the IFN-τ secretion. In contrast, to our knowledge, only *IFITM1* and *IFITM3*^35^ and not *IFITM2* have been reported to respond to the IFN-τ stimuli during early pregnancy. The *CLEC3B* also presented an expression profile similar to the classic ISGs. In this regard, transcriptomic studies in cattle reported that *CLEC3B*^36^ and also the other two genes, members of the same domain but with different families, *CLEC4F*^37^ and *CLEC2B*^34^, are stimulated by conceptus presence in the endometrium tissue. Considering these reports and the present results is suggested that *CLEC3B* is also a gene stimulated by IFN-τ on peripheral immune cells.

The TNF superfamily (*TNFSF*) was also previous related to viral response mediated by type I IFN^38,39^. In the present study, we analyzed on PBMC the expression profile of *TNFSF13B*, which was previously reported as a DEG in the endometrium on day 16 of pregnancy in heifers^34^ and in the granulocytes cultured with IFN-τ^29^. Interestingly, we observed its expression on immune cells, with an earlier increased expression on PBMC than most ISGs previously reported^14,15^. A greater expression of *TNFSF13B* was observed on D16 and D18 in the P heifers. This suggests that this gene has an earlier response than the classic ISGs and can be stimulated by INF-τ or another molecule secreted from the conceptus, but probably using a different pathway as it is a pro-apoptotic gene^40^. The *C1R* is important for the modulation of the maternal immune system, preventing the embryo rejection on bovine endometrial cells during the pre-attachment period^41^. The *C1R* was expressed in the endometrium of *Bos taurus taurus* heifers analyzed by transcriptomic analyses on day 16^34^ and 18^42^ post-estrus and by microarray analysis on day 21 of pregnancy in PMN^29^. Considering this later report and our present results on immune cells, it is suggested that the *C1R* is involved in the early immune response to conceptus presence during the pre-attachment period of pregnancy, but its use for detection of pregnancy in immune cell is limited as we only detected an up regulation of this gene on D18 in PMN.

Another interesting DEG evaluated that resulted in a potential pregnancy marker was *LGALS3BP*. Previous studies^41–43^ described the involvement of this gene during early pregnancy, but more related to the cellular adhesion process in the bovine endometrium during the pre-attachment period. In humans, the *LGALS3BP* is stimulated by type I IFN on peripheral immune blood cells^44^. The different expression profile between the two immune cell types is curious as this indicates that the PBMC response to conceptus was earlier than in PMN, which is the opposite outcome to the results previously reported by Kizaki et al.^13^. Also, a previous study^45^ reported the involvement of *LGALS2BP* with macrophage activation and mediation of immune response regulated by type I IFN and neopterin, without granulocytes involvement. The *LGALS2BP* gene may act with high intensity on cells of the monocyte-macrophage lineage^45^, which could explain the different *LGALS3BP* expression profiles between PMN and PBMC in the present study.

The *DMNK* increased abundance in PBMC on D16, also indicated that the conceptus presence stimulated its up-regulation in peripheral immune cells. This gene is abundant in stratified epithelia and in differentiating primary human keratinocytes, mainly related to inflammatory skin disorders^46^. During inflammatory disorders, the *DMKN* expression is regulated by cytokines such tumor necrosis factor α (TNFα) and interferon β (IFN-β)^46^. The IFN-β like IFN-τ is one of type I IFNs, which suggests that the IFN-τ can stimulate the *DMKN* expression in immune cells. Additionally, other studies have shown the expression of one specific isoform *DMKNα* in the mouse and humans placenta^47,48^, but without a function determined yet. Its expression was down-regulated on D18 in the present study, which indicates that this gene is a poor pregnancy marker in cattle during this period.

Although the *A2M, BPI*, *ANG*, *PLSCR2* and *DRAM1* were not affected by the pregnancy status*,* a temporal change on expression of these genes was observed. One possible explanation for this result is that the normal changes on steroid hormones during estrous cycle could have a major effect on expression of these genes rather than the pregnancy status. Therefore, the increase on plasma P4 concentrations from D10 to D16 regardless the pregnancy status group could have affected the expression of these genes. One alternative explanation regarding the up regulation on *BPI*, *ANG*, *PLSCR2* and *DRAM*1 is that all heifers were inseminated, and NP heifers may have experienced the presence of an embryo during the first 2 weeks and then underwent pregnancy loss around the period of pregnancy recognition (days 15–18), resulting in up regulation on the expression of these genes on D18. In addition, the *ANG* family gene has already been associated with the growth or demise of luteal tissue through alterations in vascularity^49^ that is necessary independent of the pregnancy status and regulated during the luteolysis around day 18 of the estrous cycle, the same moment of higher expression of *ANG* in the present study. Increased expression of *BPI* and *PLSCR2* has also been associated with the immune response of cows to bacterial and virus infection, respectively^50,51^. In this regard, the *PLSCR2* in the viral response is stimulated by activation of STAT pathway induced by type I IFN^50^, as the classic ISGs; however, a difference in the expression profile between P and NP heifers was not observed in the herein study.

**Figure 6:**
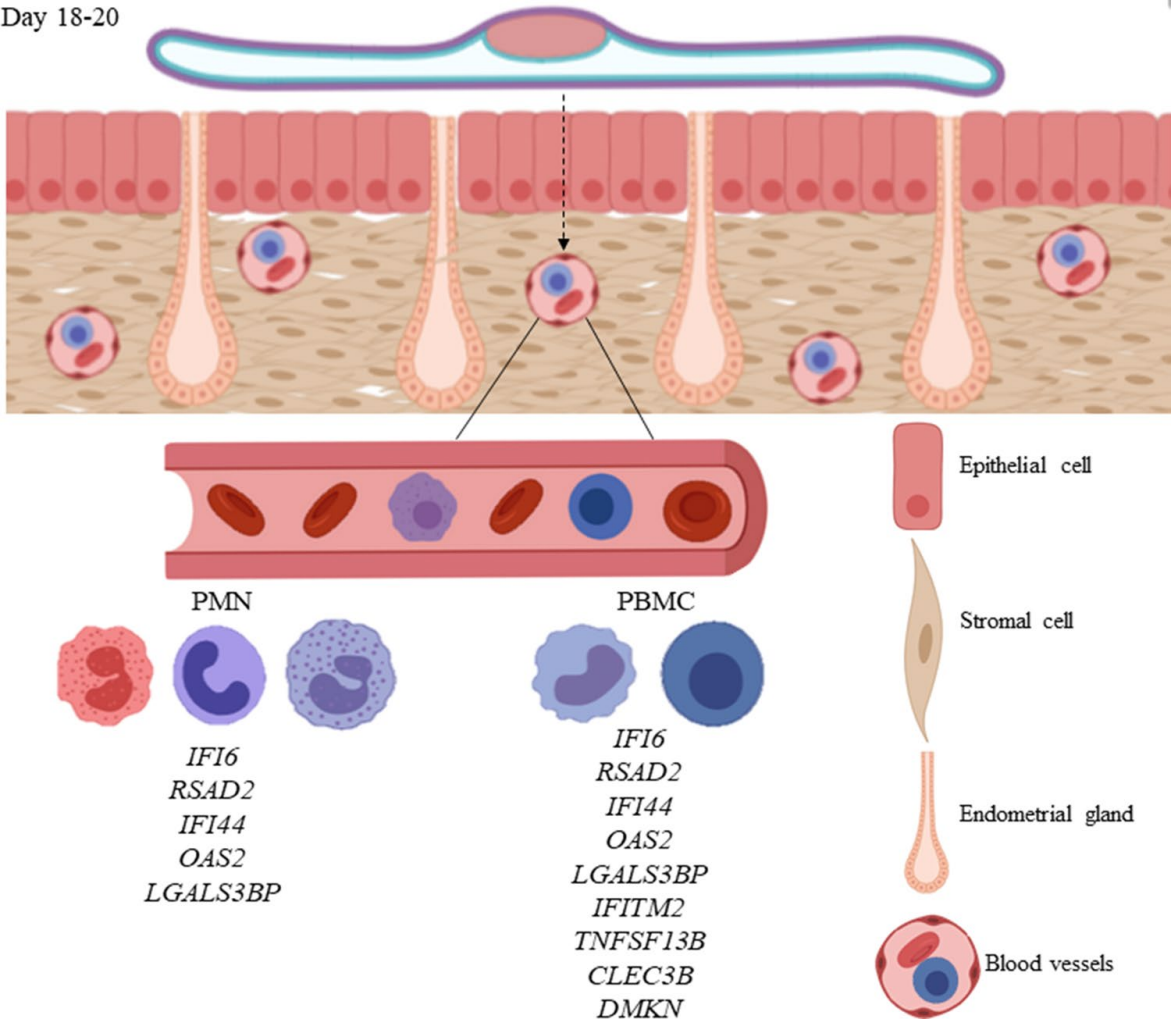
Summary and integration of the main results. Relative expression indicated that from days 18 to 20 of pregnancy, the elongate conceptus is able to stimulate the expression of the specific novel transcripts in PBMC (*IFI6, RSAD2, IFI44, OAS2, LGALS3BP, IFITM2, TNFSF13B, CLEC3B* and *DMKN*) and PMN (*IFI6, RSAD2, IFI44, OAS2* and *LGALS3BP*) in cattle. We propose that these novel transcripts may be used as markers in the bloodstream for early pregnancy detection in cattle.

In summary, the presence of a viable conceptus stimulates a large number of DEGs in peripheral leucocytes on D18 (Fig. 6). The comprehension of the temporal changes of 20 novel DEGs between P and NP beef heifers during early pregnancy allowed the discovery of nine potential pregnancy markers on PBMC (*IFI6*, *RSAD2*, *IFI44*, *OAS2*, *LGALS3BP*, *IFITM2*, *TNFSF13B*, *CLEC3B* and *DMKN*) and five on PMN (*IFI6*, *RSAD2*, *IFI44*, *OAS2* and *LGALS3BP*). The discovery of these novel early-pregnancy markers on immune cells retrieved from peripheral blood can be used for the development of new methods to predict positive pregnancies earlier than the traditional diagnostic methods. Alternatively, genes differentially expressed in both cellular types (*IFI6, RSAD2, IFI44, OAS2* and *LGALS3BP*) could be studied in total blood as an easier and faster toll to detect the pregnancy. Similarly, the present findings prompted the formulation of hypotheses regarding new molecules and mechanisms involved in early pregnancy signaling and recognition in cattle.

## Methods

### Animals

Animal welfare guidelines and handling procedures recommended by the São Paulo State (Brazil) law number 11.977 were strictly followed. That experiment was approved by the Animals Ethics Committee of the School of Veterinary Medicine and Animal Science (CEUA-FMVZ number: 6810070817). Nelore (*Bos taurus indicus*) nulliparous beef heifers (n = 29) weighing 340 kg ± 32, with a body condition score between 3 and 4 (scale 1–5), with no gross reproductive anomalies were maintained in grazing conditions and received water and mineral supplementation ad libitum. Pre-breeding examinations were performed approximately 45 days before the experiment to ensure that all heifers were pubertal (presence of CL and a developed reproductive tract), as indicated by Holm^52^.

Heifers were subjected to an estrous synchronization protocol for TAI based on P4 and estradiol treatments. Briefly, on D–10 (D0 = TAI) an intravaginal P4 device (1.0 g of P4; Sincrogest; Ourofino Saúde Animal, Cravinhos, SP, Brazil) was inserted, and 1 mL i.m. of estradiol benzoate (2 mg; Sincrodiol; Ourofino Saúde Animal) and 2 mL i.m. of PGF2α (500 µg; of sodium cloprostenol; Sincrocio; Ouro Fino Saúde Animal) were injected. Eight days later, the P4 device was withdrawn and the animals were treated i.m. with a second PGF2α dose (Sincrocio; Ouro Fino Saúde Animal), 1 mL of estradiol cypionate (1 mg; Sincro CP; Ourofino Saúde Animal) and 1.5 mL of equine chorionic gonadotropin (300 U.I; Sincro eCG; Ouro Fino Saúde Animal). Two days after (D0), all heifers were treated i.m. with a gonadotropin-releasing hormone analog (10 µg; of buserelin acetate; Sincroforte; Ouro Fino Saúde Animal) and were artificially inseminated by a single operator using thawed semen from a single Nelore sire.

On D10, D14, D16, D18 and D20, blood samples (40 mL) were collected from the jugular vein into evacuated tubes (BD Vacutainer; São Paulo, Brazil) containing sodium heparin for isolation of immune cells and evaluation of plasma P4 concentration. On the same days, B-mode and color Doppler ultrasonography exams were performed to measure the area and blood perfusion of CL, respectively. Pregnancy diagnostics were performed on D28 and heifers were assigned to the pregnant (P) or non-pregnant (NP) groups, based on the presence or absence of an embryo with heartbeat.

### Isolation of immune cells

Blood samples collected for PBMC and PMN isolation (≈ 30 mL) were submitted to a Ficoll gradient protocol. To isolate each group of cells, whole blood was mixed with an equal volume of PBS, and the solution was layered onto 15 mL of Ficoll-Paque solution (GE Healthcare, Uppsala, Sweden), placed in a 50 mL conical tube, and then centrifuged at 1,100×*g* for 30 min 20 °C to obtain the buffy coat, as described previously^14^. After the gradient formation, the buffy coat was utilized for PBMC isolation and the last layer containing the granulocytes and red blood cells was utilized for PMN isolation, as reported previously^53^. The PBMC and PMN were subject to successive lyses steps with hypertonic solutions and then PBS restored the isotonicity. The resulting pellet was stored in a 1.5 mL conical tube at − 80 °C until RNA extraction. To check the purity of the PBMC and PMN, freshly isolated samples of each cell type were placed on a slice and stained with fast panotic method for morphological identification of cells by light microscopy under 400× magnification. The purity > 95% for all samples.

### Plasma progesterone concentrations

For P4 analysis, blood samples were stored in a box with chopped ice immediately after collection, plasma was harvested by centrifugation (2700 g/15 min/4 °C) and stored at − 20 °C. Concentrations of P4 were assayed with a solid-phase Radioimmunoassay kit (ImmuChem coated tube, MP Biomedicals, Costa Mesa, USA). The intra-assay CV for high and low reference controls and sensitivity for P4 were respectively, 2.32%, 2.03% and 0.01 ng/mL.

### Ultrasound evaluations

Ultrasound scanning was performed at D0 to detect a dominant follicle and 48 h later to confirm ovulation. On D10, 14, 16, 18 and 20, ultrasonography evaluations were executed to measure the area and blood perfusion of CL using the color Doppler mode. A duplex B-mode (gray-scale) and pulse-wave color Doppler ultrasound instrument (MyLab Delta Vet Gold; Esaote Healthcare; Italy) equipped with a multifrequency linear transducer (3.5–7.5 MHz) in B mode (RES-A, gain 50%, P 74 mm, X/M, PRS 1) and Doppler mode (gain 61%, PRF 730 Hz, frequency 6.3 MHz, WF 4, PRS 3, PRC M/2) was used. Area of the CL was determined using a B-mode still image and the tracing function. For CLs with anechoic fluid-filled cavities, the area of the cavity was subtracted from the total area^54^. The percentage of luteal area with color Doppler signals of blood flow at each examination was determined as described previously^14,55^.

### Study 1: discovery of novel pregnancy markers

To discover novel pregnancy markers, PBMC cells collected on D18 from six P and six NP heifers submitted to RNAseq. Although the minimum number of replicates commonly used for reliable results in RNAseq experiments is three per group, we preferred to increase it to six per group to be more robust and reduce the chance of false positive results^56^. Females were selected based on the CL blood perfusion and area on D18 (heifers bearing the largest CLs with at least > 40% of blood perfusion for the P group, and heifers bearing the smallest CLs with < 10% of blood perfusion for the NP group). These criteria was expected to generate contrasting groups of animals as the CL blood perfusion and area are strong correlate with P4 concentrations^54^, which is required during the pregnancy.

#### RNA extraction, library preparation, and RNA sequencing

The PBMC samples selected for RNAseq (n = 6/group), were submitted to RNA extraction. Cell pellets were thawed and immediately mixed with the lysis buffer from the RNeasy Mini Column Kit (QIAGEN, Hilden, Germany), according to the manufacturer’s instructions. The RNA yield was quantified using the Qubit RNA Broad Range Assay Kit (Eurogene, USA) on a Qubit Fluorometer, and integrity was assessed on Agilent 2,100 Bioanalyzer (Agilent Technologies, Santa Clara, USA) using an Agilent RNA 6,000 Nano chip (Bioanalyzer, Agilent, Santa Clara, USA). All Samples used for RNAseq obtained RIN values ranging between 7.1 and 9.4. Libraries were prepared with 1 µg of RNA in the TruSeq Stranded mRNA Library Prep kit (Illumina, San Diego, USA) following manufacturer’s instructions. The Library size distribution was estimated through the Agilent DNA 1,000 chip (Agilent Technologies) and the library concentration was measured through Quantitative Real-Time PCR (qPCR) with a KAPA Library Quantification kit (KAPA Biosystems, Basileia, Switzerland). Samples were diluted, pooled in equimolar amounts and then sequenced at the “Centro Genômico Funcional Aplicado a Agropecuária e Agroenergia” at the University of São Paulo (São Paulo-SP, Brazil) using a HiSeq 2,500 Sequencer (Illumina).

#### Statistical analyses

Statistical analyses of P4 concentrations and CL characteristics were performed using SAS (version 9.2, SAS Institute Inc., Cary, NC, USA). Heifer was considered the experimental unit. Data were evaluated for the detection of outliers using Dixon test and for the normal distribution according to the Shapiro–Wilk test. When the raw data did not follow a normal distribution, they were transformed into natural logarithms or ranks. The plasma P4 concentrations, area, and blood perfusion of CL on D18 were analyzed using ANOVA by the MIXED procedure (SAS), considering the random effect of heifer.

#### Bioinformatics analyses

The sequences generated were filtered to remove low-quality sequences and contaminated reads using the SeqyClean 1.9.9 (https://github.com/ibest/seqyclean), and only high-quality paired-end sequences (with average PhredScore over 24) were used for further analysis. The samples were mapped against the *Bos taurus taurus* genome (ARS-UCD1.2) using the function –quantMode from the package STAR^57^. The significance of differential gene expression was assessed with the edgeR program^58^. These analyses were run in R/Bioconductor^59^, for each comparison analyses were conducted separately. Count data were first normalized by trimmed mean of M-values (TMM)^60^ for differences in sequencing effort and proportionality across libraries using the calcNormFactors function, while common dispersions were calculated using the estimate CommonDisp function^61^^.^ A Benjamini–Hochberg correction^62^ for multiple tests was applied to avoid false positives. Only genes with FDR < 0.05 were considered significant differentially expressed.

#### Enrichment analyses

The list of differentially expressed genes (DEGs), was analyzed using DAVID Bioinformatics Resources^63^ for gene enrichment analysis relying on *Bos taurus* annotation from the Gene Ontology (GO) consortium (geneontology.org). KEEG pathways were also assessed through the DAVID Bioinformatics Resources^63^. Both enrichment analyses adopt the Hypergeometric Test along with the Benjamini & Hochberg *p* value adjustment method^62^.

### Study 2: Expression profile of the novel markers in immune cells

**Figure 7:**
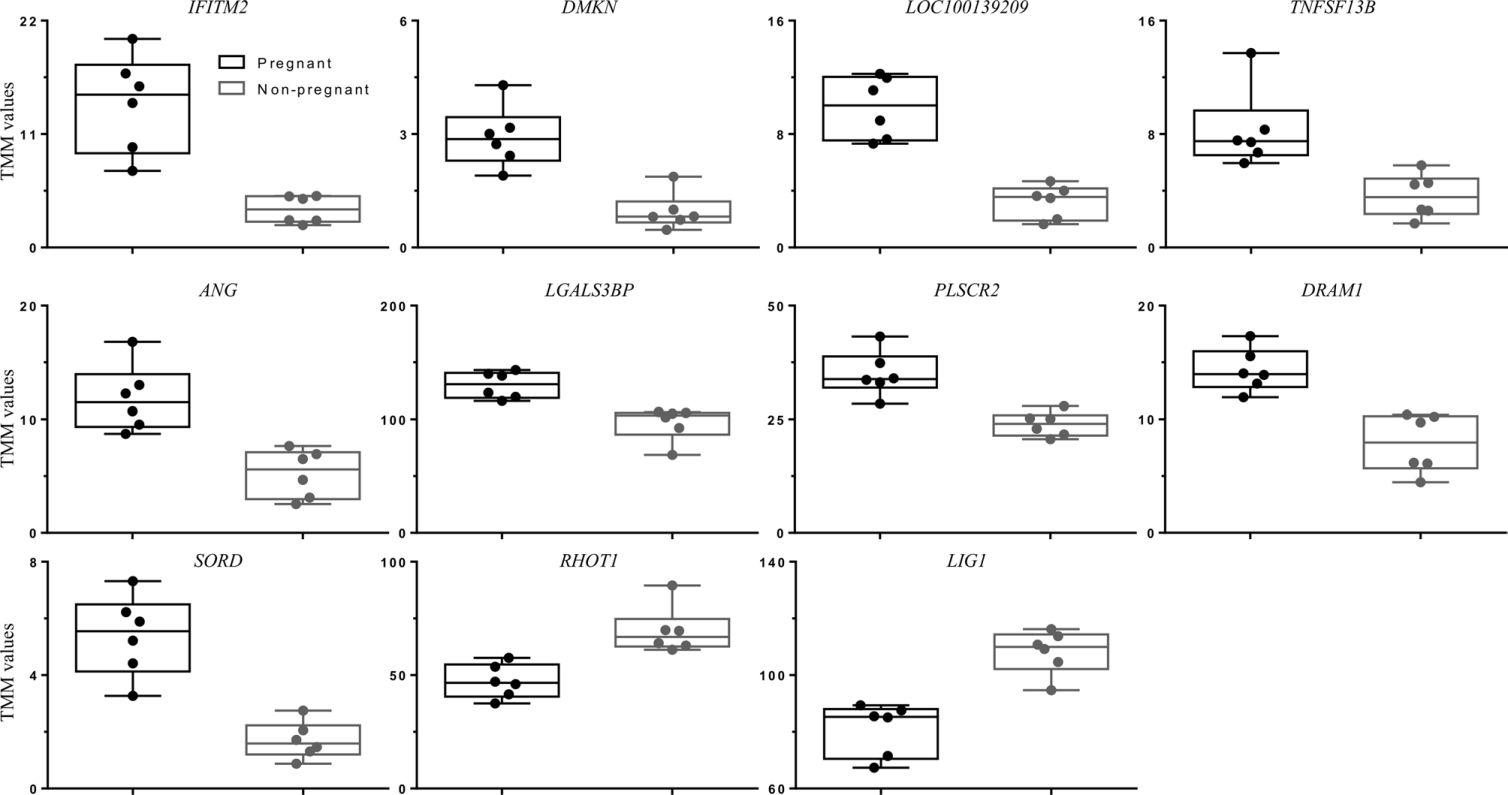
Box plot showing the maximum and minimum of trimmed mean of M-values (TMM) for the eleven genes with absence of overlap evaluated on qPCR. Each circle represents the specific TMM of each animal (n = 6/group).

The expression of 20 novel transcripts, that were identified as differentially expressed between P and NP groups in Study 1 was evaluated in PBMC and PMN samples collected on D10, 14, 16, 18, and 20. Twelve heifers were selected randomly from the P and NP groups (n = 6/group). Therefore, some heifers (n = 3) were also used for the transcriptome analysis in Experiment 1. The criteria used to select the genes discovered in Study 1 were: (1) differentially expressed genes (DEGs) that did not present overlap in the RNAseq TMM values between NP and P groups (Fig. 7); and (2) the DEGs with the highest log fold change (logFC) values.

#### RNA extraction, cDNA synthesis, and quantitative real-time reverse transcriptase polymerase chain reaction (qPCR)

Total RNA extraction of PBMC was performed using TRIzol reagent (TRIzol reagent; Life Technologies; Frederick, USA) in accordance with manufacturer’s guidelines. The PMN samples were submitted to RNA extraction through TRIzol and chloroform gradient, after the RNA obtaining the samples were passed through a column of the kit mini prep Direct-zol (ZYMO Research, USA) for purification in accordance to manufacturer’s guidelines. During the PMN extraction, the RNA was treated with DNase I included in the kit (mini prep Direct-zol) for 15 min at room temperature. Total RNA extracted from both cells was separately eluted with 20 µL of RNAse free water. Concentrations and purity of RNA in extracts were evaluated using spectrophotometry (NanoVueTM Plus Spectrophotometer, Ge Healthcare. UK) and absorbance ratios values (260/280) ranged between 1.7 and 2.

Before the reverse transcription, the isolated RNA from PBMC was treated with DNase I (DNaseI, Amplification Grade; Life Technologies) for genomic DNA contamination as per manufactures instructions. We synthesized complementary DNA (cDNA) from 500 ng (PMN) and 1000 ng (PBMC) of total RNA using the High-Capacity cDNA Reverse Transcription Kit (Life Technologies). A master mix (10 µL) containing random primers, reverse transcriptase enzymes and deoxynucleotides were added to 11 µL of samples. Samples were incubated at 25 °C for 10 min and then at 37 °C for 2 h, subjected to reverse transcriptase inactivation at 85 °C for 5 min, and stored at − 20 °C until qPCR analysis. The final RT reaction was diluted 1:80 and this cDNA was used as a template for each qPCR reaction.

Analyses of relative abundance of transcripts were performed using SYBR Green PCR Master Mix (Life Technologies) for the amplification reactions in a Step One Plus thermocycler (Applied Biosystems Real-Time PCR System; Life Technologies). Samples were run in triplicate and the maximum CV accepted among the replicates was 0.1. The optimized primer pairs were designed using primer design platform from national center of biotechnology information (NCBI) (https://www.ncbi.nlm.nih.gov/tools/primer-blast) based on the mRNA sequence of target genes obtained from the RefSeq database, on Genbank (https://www.ncbi.nlm.nih.gov/genbank/) and the specificity of the primer were checked by BLAST (NCBI, https://blast.ncbi.nlm.nih.gov/Blast.cgi). The qPCR products were submitted to SANGER-DNA sequencing, and identities of target genes were confirmed. Details of primers are provided in Table 3. In order to select reference genes, the Normfinder Microsof Excel applet was used^64^. The Glyceraldehyde-3-Phosphate Dehydrogenase (*GAPDH*) and Actin Beta (*ACTB*) were the most stable genes in PMN and *GAPDH* and Ciclofilin (*PPIA*) were the most stable genes on PBMC, therefore, were selected as reference genes among the others evaluated (Ribosomal Protein L30 [*RPL30*], Ribosomal Protein L15 [*RPL15*] and 18 S Ribosomal RNA [*18S*]).

**Table 3:** Bovine specific oligonucleotide forward and reverse primer sequences (5′-3′), primer efficiency in the standard curve and amplicon length of the genes evaluated on qPCR.

Target name	Gene number	Forward primer sequence (5′-3′)	Reverse primer sequence (5′-3′)	Efficiency (%)	Length (bp)
*A2M*	NM_001109795.1	TGCAACACAGTCTGGTCTCC	AGCACCATGTATTGCGGTTTT	107	143
*ANG*	NM_001078144.1	GGCCGAGGAGCCTTTGTTG	CATTCCGGCCCTTTGGTTTG	95	164
*BPI*	NM_173895.2	GCTGCCAGTGACAACCAAAC	TACACCATGCGATCGTGGTC	95	189
*CLEC3B*	NM_001046212.1	TGCCAAGAAAGATGCTGTGAG	TGGAAGGTCTTCGCTTGGAC	94	177
*CR1*	NM_001034407.1	GGCCTTGAGAAATGTGGCTCT	AGGGTAAGGCTTGGGGAACA	109	128
*DMKN*	NM_001082463.1	CAGGATGGCAAGACGCAGTA	CGGAAGTGCTTGCCTACCAA	91	192
*DRAM1*	NM_001031767.2	TGTACACGCTCCTGCAATCC	GAAGCACAGGCAATCATGGG	97	133
*IFI44*	XM_002686295.6	TCTGCCCATTGCTGAAGGAC	CCACATGGACCACATCAGACT	97	141
*IFI6*	NM_001075588.1	TGCTCTCCTCCAAGATACGGT	CAGAAGCTCGAGTCGCTGTT	106	161
*IFITM2*	NM_001078054.2	GCACATCGATCTCCCTACACA	GGTGTTGAACAGGGACCACA	104	157
*LGALS3BP*	NM_001046316.2	GGACTCGAGGCGTGAAAGAC	ATGTTCTCACACACCGTCCC	93	112
*LIG1*	NM_001102548.1	AACGGAAAGTCCCTGGTACG	TCACCGACTGCTCCAAGAAC	92	151
*LOC100139209*	XM_024989137.1	CTCCATCCACTACATGGGGC	CACTGGAGGGTCACATTCCC	98	183
*OAS2*	NM_001024557.1	GATCCCACTGACCCAACCAA	GTGATGCAGGCAGAACATTCC	95	139
*PLSCR2*	NM_001034436.1	GTGTACCAAAGAAGGACACACT	ACACTTGAGGGATCTGAAAACT	97	152
*RHOT1*	NM_001046082.1	CGTAGCTGCAAAGTCAGACC	CTTGTGTCACGTGCGGGTA	96	182
*RSAD2*	NM_001045941.1	TGGTTCCAGAAGTACGGTGAA	ACCACGGCCAATAAGGACAT	97	90
*SIGLEC1*	XM_025001078.1	GAGACAGCGGCACTTCGAG	GGTTCTGTTGCCTTGTTCTTTG	93	187
*SORD*	NM_001037320.1	CGGCATCTACGCCACTCATT	GGGTGACCAAGGGCTTTACA	95	194
*TNFSF13B*	NM_001114506.1	CTGCCCTGAAACAGCGGAT	CGTATAGTGGGCGTGTCACT	99	154

Determination of qPCR efficiency and Cq (quantification cycle) values per sample were performed with LinRegPCR software. Quantification was obtained after normalization of the target genes expression values (Cq values) by the geometric mean of the endogenous control expression values^65^. Twenty target genes were evaluated on PBMC cells. For PMN, genes with an increased expression in P or NP groups in PBMC were selected and only those validated in this immune cell type were evaluated (n = 9).

#### Statistical analyses

The data were evaluated for detection of outliers using Dixon test and the significant (*P* < 0.05) outliers detected were excluded from the analyses. The data that were not normally distributed according to the Shapiro–Wilk test were transformed with natural logarithm, rank and square root. The plasma P4 concentrations, area and blood perfusion of CL and the abundance of each transcript were analyzed by split-plot ANOVA using the MIXED procedure of SAS software (Version 9.2; SAS Institute) with a REPEATED statement to account for the autocorrelation between sequential measurements. The main effects of group (P or NP) and time (D10, 14, 16, 18 or 20) and their interaction (group-by-time) were evaluated for each variable, considering the significant (*P* ≤ 0.05) and approached significant effects (*P* ≤ 0.1). Heifers-within-group was used as a random effect. For the ratio and difference between D18 and D10 we considered each heifer as an experimental unit, using ANOVA by the MIXED procedure (SAS), heifer as a random effect, considering the significant (*P* ≤ 0.05) and approached significant effects (*P* ≤ 0.1).

## Supplementary information

Supplementary file1

Supplementary file2

Supplementary file3

Supplementary file4

## Data Availability

The data discussed in this publication have been deposited in NCBI’s Gene Expression Omnibus (GEO) and are accessible through GEO series accession number GSE136102. (https://www.ncbi.nlm.nih.gov/geo/query/acc.cgi?acc=GSE136102).
