# Supplementary figures and images for: Early pregnancy-induced transcripts in peripheral blood immune cells in Bos indicus heifers
Source: Sci Rep. 2020 Aug 13;10:13733. doi: 10.1038/s41598-020-70616-8 (PMC7426272; doi:10.1038/s41598-020-70616-8)

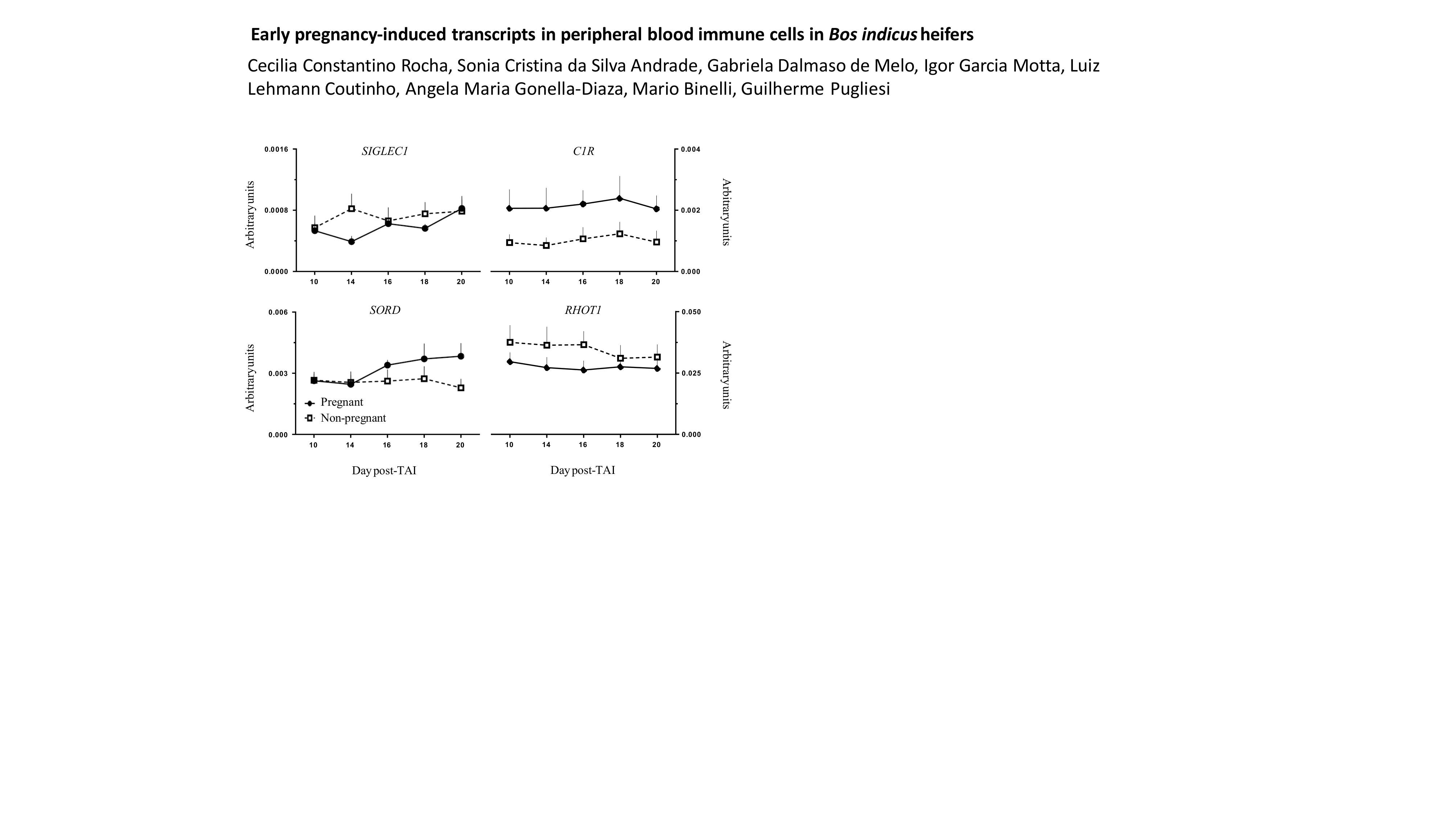

Supplement: Supplementary file 3 — Supplementary file3 [file 41598_2020_70616_MOESM3_ESM.tif]

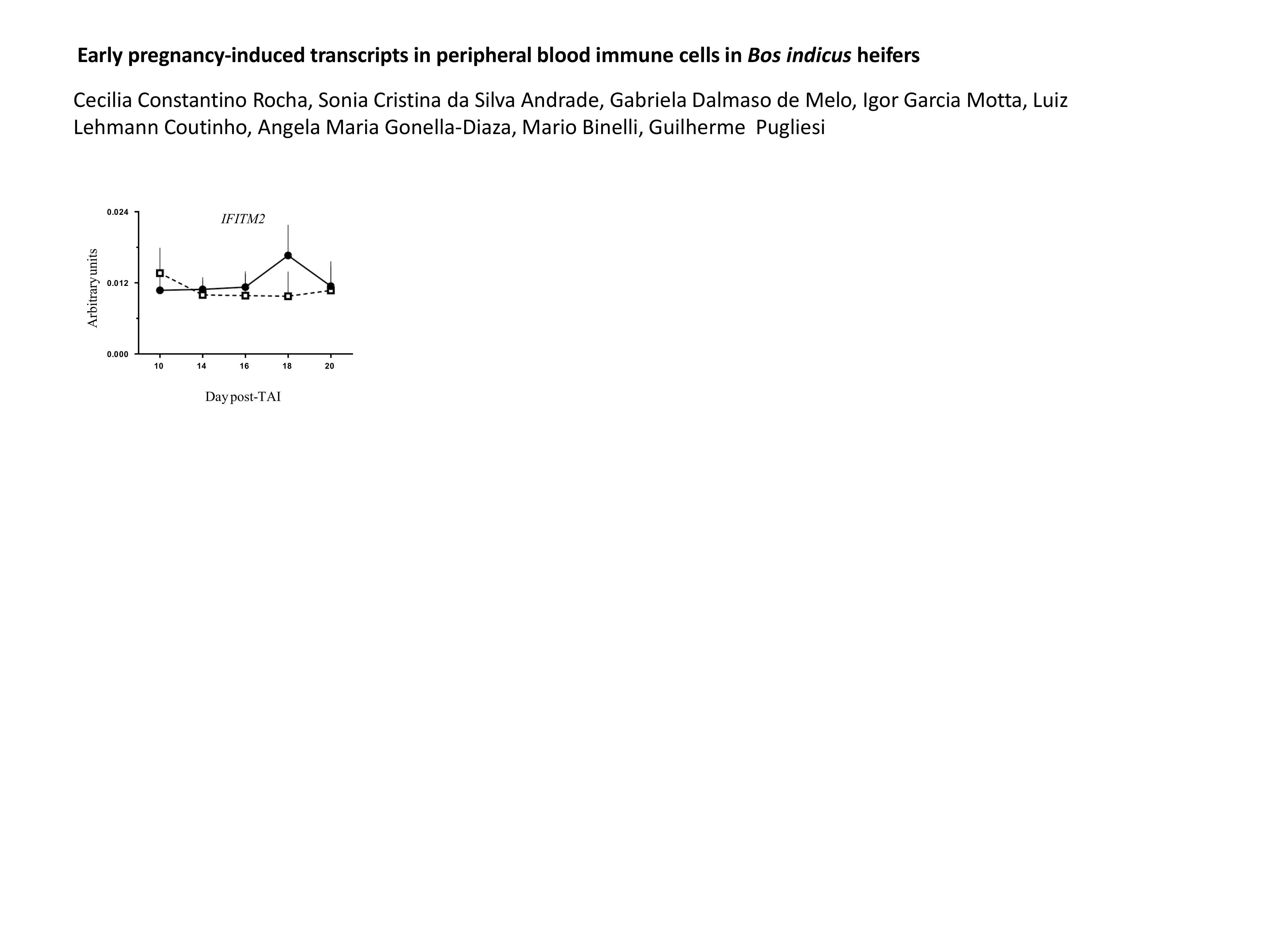

Supplement: Supplementary file 4 — Supplementary file4 [file 41598_2020_70616_MOESM4_ESM.tif]
